# Comparative analysis of the complete chloroplast genomes of six threatened subgenus *Gynopodium* (*Magnolia*) species

**DOI:** 10.1186/s12864-022-08934-6

**Published:** 2022-10-20

**Authors:** Huanhuan Xie, Lei Zhang, Cheng Zhang, Hong Chang, Zhenxiang Xi, Xiaoting Xu

**Affiliations:** 1 Key Laboratory of Bio-Resource and Eco-Environment of Ministry of Education, College of Life Sciences, Sichuan University, Chengdu, 610065 China; 2 Key Laboratory of Ecological Protection of Agro-Pastoral Ecotones in the Yellow River Basin National Ethnic Affairs Commission of the People’s Republic of China, College of Biological Science & Engineering, North Minzu University, Yinchuan, 750021 China; 3 Chengdu Institute of Biology, Chinese Academy of Sciences, Chengdu, 610041 China

**Keywords:** Threatened species, Subgenus *Gynopodium*, Chloroplast genomes, Comparative genomics, Nucleotide diversity, Phylogenomics

## Abstract

**Background:**

The subgenus *Gynopodium* belonging to genus *Magnolia* have high ornamental, economic, and ecological value. Subgenus *Gynopodium* contains eight species, but six of these species are threatened. No studies to date have characterized the characteristics of the chloroplast genomes (CPGs) within subgenus *Gynopodium* species. In this study, we compared the structure of CPGs, identified the mutational hotspots and resolved the phylogenetic relationship of subgenus *Gynopodium*.

**Results:**

The CPGs of six subgenus *Gynopodium* species ranged in size from 160,027 bp to 160,114 bp. A total of 131 genes were identified, including 86 protein-coding genes, eight ribosomal RNA genes, and 37 transfer RNA genes. We detected neither major expansions or contractions in the inverted repeat region, nor rearrangements or insertions in the CPGs of six subgenus *Gynopodium* species. A total of 300 large repeat sequences (forward, reverse, and palindrome repeats), 847 simple sequence repeats, and five highly variable regions were identified. One gene (*ycf1*) and four intergenic regions (*psbA-trnH-GUG*, *petA*-*psbJ*, *rpl32-trnL-UAG,* and *ccsA-ndhD)* were identified as mutational hotspots by their high nucleotide diversity (*Pi*) values (≥ 0.004), which were useful for species discrimination. Maximum likelihood and Bayesian inference trees were concordant and indicated that Magnoliaceae consisted of two genera *Liriodendron* and *Magnolia*. Six species of subgenus *Gynopodium* clustered as a monophyletic clade, forming a sister clade with subgenus *Yulania* (BS = 100%, PP = 1.00). Due to the non-monophyly of subgenus *Magnolia*, subgenus *Gynopodium* should be treated as a section of *Magnolia*. Within section *Gynopodium*, *M. sinica* diverged first (posterior probability = 1, bootstrap = 100), followed by *M. nitida, M. kachirachirai* and *M. lotungensis*. *M. omeiensis* was sister to *M. yunnanensis* (posterior probability = 0.97, bootstrap = 50).

**Conclusion:**

The CPGs and characteristics information provided by our study could be useful in species identification, conservation genetics and resolving phylogenetic relationships of Magnoliaceae species.

**Supplementary Information:**

The online version contains supplementary material available at 10.1186/s12864-022-08934-6.

## Background

The genus *Magnolia* is one of the early diverged angiosperm lineages consisting of approximately 300 species across three subgenera: *Gynopodium*, *Magnolia*, and *Yulania* according to Figlar’s taxonomic system [1, 2]. The extensive changes in chromosome number and rare androdioecious flowers of subgenus *Gynopodium* make them important materials for studying the evolution and breeding of flowering plants, as they are thought to represent a key transition from bisexual flowers to unisexual flowers [3–5]. Furthermore, members of the subgenus *Gynopodium* are known for their beautiful flowers, leafy branches, and aesthetically appealing shapes having high ornamental, economic, and ecological value [6, 7]. However, over-harvesting coupled with weak regenerative capacity makes the wild populations of subgenus *Gynopodium* species decreased rapidly [8–10]. Six of the eight subgenus *Gynopodium* species are of conservation concern, including three critically endangered species, two endangered species, and one vulnerable species according to the IUCN Red List [11]. Despite lots of studies on phytocoenological characteristics and breeding of subgenus *Gynopodium* [8, 9, 12], investigations of the genomic characteristics of this subgenus remain lacking.

Compared with the nuclear genome, the chloroplast genome (CPG) has a small size, low nucleotide substitution rate, single-parental inheritance, and haploid nature, which make it a good option for the analyses of nucleotide diversity and reconstructing phylogenies of closely related species, especially among polyploid taxa [13–15]. Although the structure of the CPG is generally conserved consisting of a large single-copy (LSC) region, a small single-copy (SSC) region, and two inverted repeat regions (IR) [16], some structural rearrangements have been discovered, including the loss of genes or introns, as well as IR expansions and contractions [17, 18]. The comparative and phylogenetic analyses of CPGs have proved an ideal tool for species identification [19], detecting structural variation [20], assessing nucleotide diversity [21], resolving phylogenetic relationships [22], and reconstructing the evolutionary history [23]. Due to the similarity in the morphology of subgenus *Gynopodium* species and the complexity of their nuclear genomes associated with polyploidy [24], the CPG is suitable for exploring phylogenetic relationships, discriminating species and providing useful information for developing conservation strategies for this subgenus [25].

Here, we used the four newly sequenced CPGs of *Magnolia omeiensis*, *Magnolia nitida*, *Magnolia sinica*, and *Magnolia kachirachirai*, in addition to two previously published CPGs of *Magnolia lotungensis* and *Magnolia yunnanensis*, to (i) characterize the structural features and variations of the CPGs for the six sugenus *Gynopodium* species, (ii) assessing nucleotide diversity and identify hypervariable regions to developing DNA markers for species discrimination and conservation genetics studies, and (iii) resolve the evolutionary relationships of subgenus *Gynopodium* species.

## Results

### Characteristics of the CPGs

**Fig. 1:**
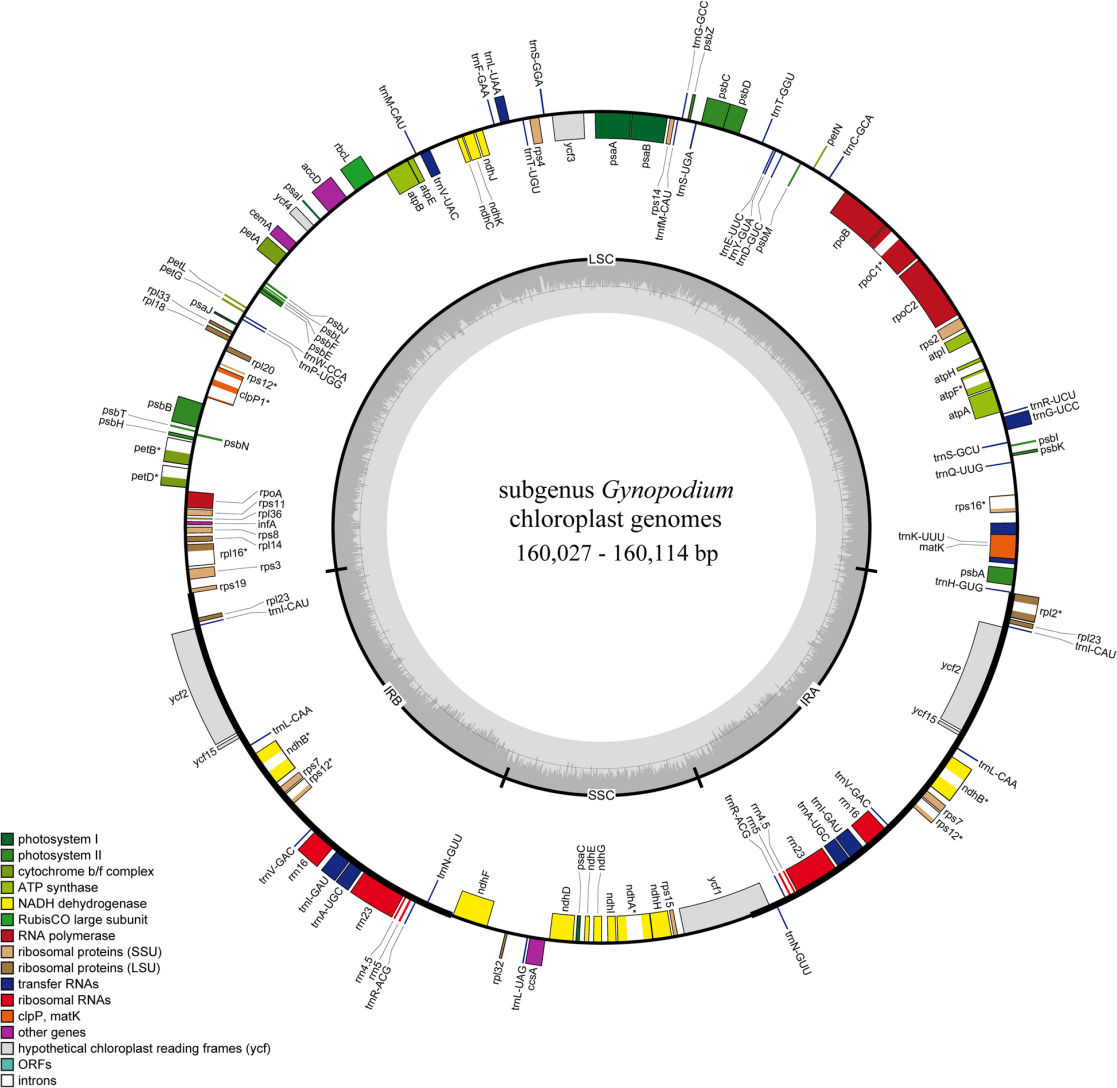
Gene map of the CPGs of six subgenus *Gynopodium* species. The genes inside and outside of the circle are transcribed in the clockwise and counterclockwise directions, respectively. Genes belonging to different functional groups are shown in different colors. The darker gray area in the inner circle indicates the GC content and the lighter gray indicates the AT content of the genome. The thick lines indicate the extent of the inverted repeats (IRa and IRb) that separate the genomes into the small single-copy (SSC) and large single-copy (LSC) regions

In this study, the coverage depth of each organelle genome reached over 100 × (*Magnolia omeiensis*: 168 × , *M. sinica*: 102 × , *M. nitida*: 132 × , *M. kachirachirai*: 103 ×). The six CPGs within the subgenus *Gynopodium* ranged in size, from 160,027 bp (*M. kachirachirai*) to 160,114 bp (*M. lotungensis*) (Table 1). All CPGs were a typical quadripartite circular structure (Fig. 1) that included a LSC region and a SSC region divided by a pair of IR regions (Fig. 1 and Table 1). The length of the LSC region ranged from 88,130 bp (*M. kachirachirai*) to 88,170 bp (*M. yunnanensis*), and the length of the SSC and IR regions ranged from 18,725 bp (*M. kachirachirai*) to 18,767 bp (*M. lotungensis*), and from 26,571 bp (*M. sinica*) to 26,586 bp (*M. kachirachirai*), respectively (Table 1). The GC-content was similar in all six CPGs. The GC content of the whole plasmid sequence was 39.3%; the GC content of the IR regions was 43.2%, which was higher than that of in LSC and SSC regions (38% and 34.3%) (Table S1). In addition, 131 genes were annotated in all six CPGs, including 37 transfer RNA (tRNA) genes, 8 ribosomal RNA (rRNA) genes, and 86 protein-coding genes (Fig. 1 and Table 1). There were two copies for seven of the protein-coding genes, seven of the tRNA genes, and four of the rRNA genes; the other 95 genes were all represented by single copies. Eleven genes possessed introns: *rps16, rps12, ropC1, rpl2, rpl16, petB, petD, ndhB, ndhA, clpP1,* and *atpF* (Table 2).

**Table 1:** Summary of the CPG features of subgenus *Gynopodium*

Items	M. omeiensis*	M. sinica*	M. nitida*	M. kachirachirai*	*M. lotungensis*	*M. yunnanensis*
Total Size (bp)	160,091	160,043	160,086	160,027	160,114	160,085
LSC (bp)	88,160	88,155	88,153	88,130	88,179	88,170
SSC (bp)	18,763	18,746	18,763	18,725	18,767	18,745
IR (bp)	26,584	26,571	26,585	26,586	26,584	26,585
GC (%)	39.30%	39.30%	39.30%	39.30%	39.30%	39.30%
Gene number	131	131	131	131	131	131
PCG	86	86	86	86	86	86
tRNA	37	37	37	37	37	37
rRNA	8	8	8	8	8	8

Species with newly sequenced chloroplast genomes are marked with asterisks

*LSC* Large single-copy, *SSC* Small single-copy, *IR* Invert repeat, *GC* Guanine-cytosine, *PCG* Protein-coding gene

**Table 2:** Gene list in the CPGs of six subgenus *Gynopodium* species

Category	Group of Genes	Gene Names	Number
Self-replication	RNA polymerase	rpoA, rpoB, rpoC1*, rpoC2	4
	Ribosomal proteins (SSU)	*rps2, rps3, rps4, rps7* (× 2*), rps8, rps11, rps12* (× 2)*, rps14, rps15, rps16*, rps18, rps19	14
	Ribosomal proteins (LSU)	*rpl2* (× 2)*, rpl14, rpl16*, rpl20, rpl23 (× 2)*, rpl32, rpl33, rpl36*	10
	Transfer RNAs	37 tRNAs (7 in the IRs (× 2))	37
	Ribosomal RNAs	*rrn4.5* (× 2), *rrn5* (× 2), *rrn16* (× 2), *rrn23* (× 2)	8
Photosynthesis	Photosystem I	*psaA, psaB, psaC, psaI, psaJ*	5
	Photosystem II	*psbA, psbB, psbC, psbD, psbE, psbF, psbH, psbI, psbJ, psbK, psbL, psbM, psbN, psbT, psbZ*	15
	Cytochrome b/f complex	petA, petB*, petD*, petG, petL, petN	6
	ATP synthase	atpA, atpB, atpE, atpF*, atpH, atpI	6
	NADH dehydrogenase	ndhA*, ndhB (× 2)*, ndhC, ndhD, ndhE, ndhF, ndhG, ndhH, ndhI, ndhJ, ndhK	12
	RubisCO large subunit	*rbcL*	1
Other genes	-	*ycf1, ycf2* (× 2)*, ycf3, ycf4, ycf15* (× 2)	7
		accD, clpP*, matK, ccsA, cemA, infA	6

^*^ indicates genes containing introns; (× 2) indicates the genes are repeated twice; The *rps12* gene is a trans-spliced gene

### Comparative analysis of CPGs

**Fig. 2:**
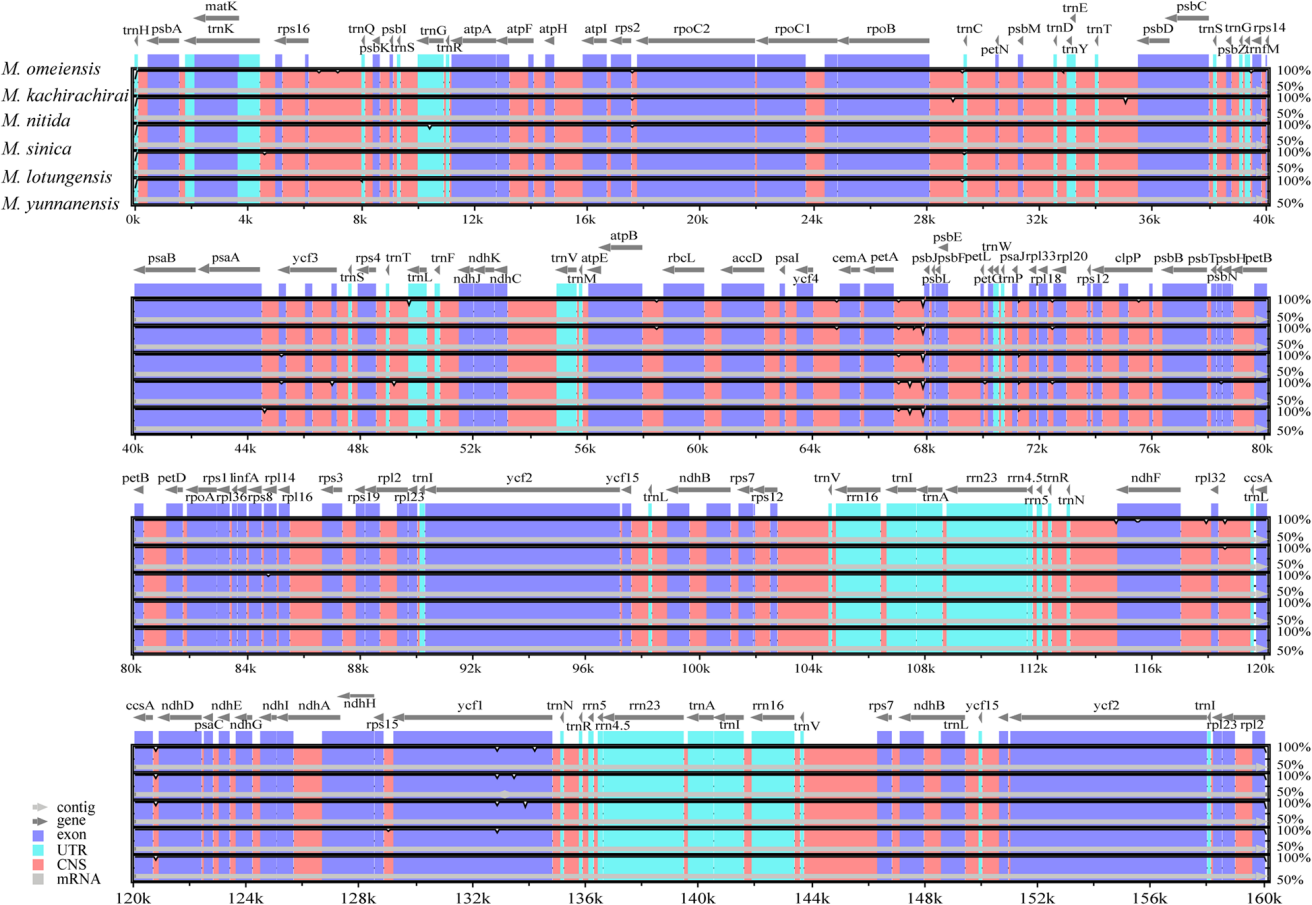
Sequence alignment of the CPGs of six subgenus *Gynopodium* species. The alignment was performed using the mVISTA program and the *M. omeiensis* chloroplast genome was used as a reference. The y-axis indicated the degree of identity ranging from 50 to 100%. Coding and non-coding regions were marked in blue and red, respectively. Black arrows indicated the position and direction of each gene. CNS: conserved non-coding sequences

The alignments indicated high sequence similarity among the CPGs of the six subgenus *Gynopodium* species. However, sequence divergence in non-coding regions was greater than that in coding regions, such as *trnH*-*psbA*, *rps2*-*rpoC2*, *ycf4*-*cemA*, *petA*-*psbJ*, and *ccsA*-*ndhD* (Fig. 2). The greatest variation among coding regions was observed in *ycf1*. No major genomic rearrangements or insertions were detected among the six CPGs relative to that of *M. omeiensis* (Fig. S1).

**Fig. 3:**
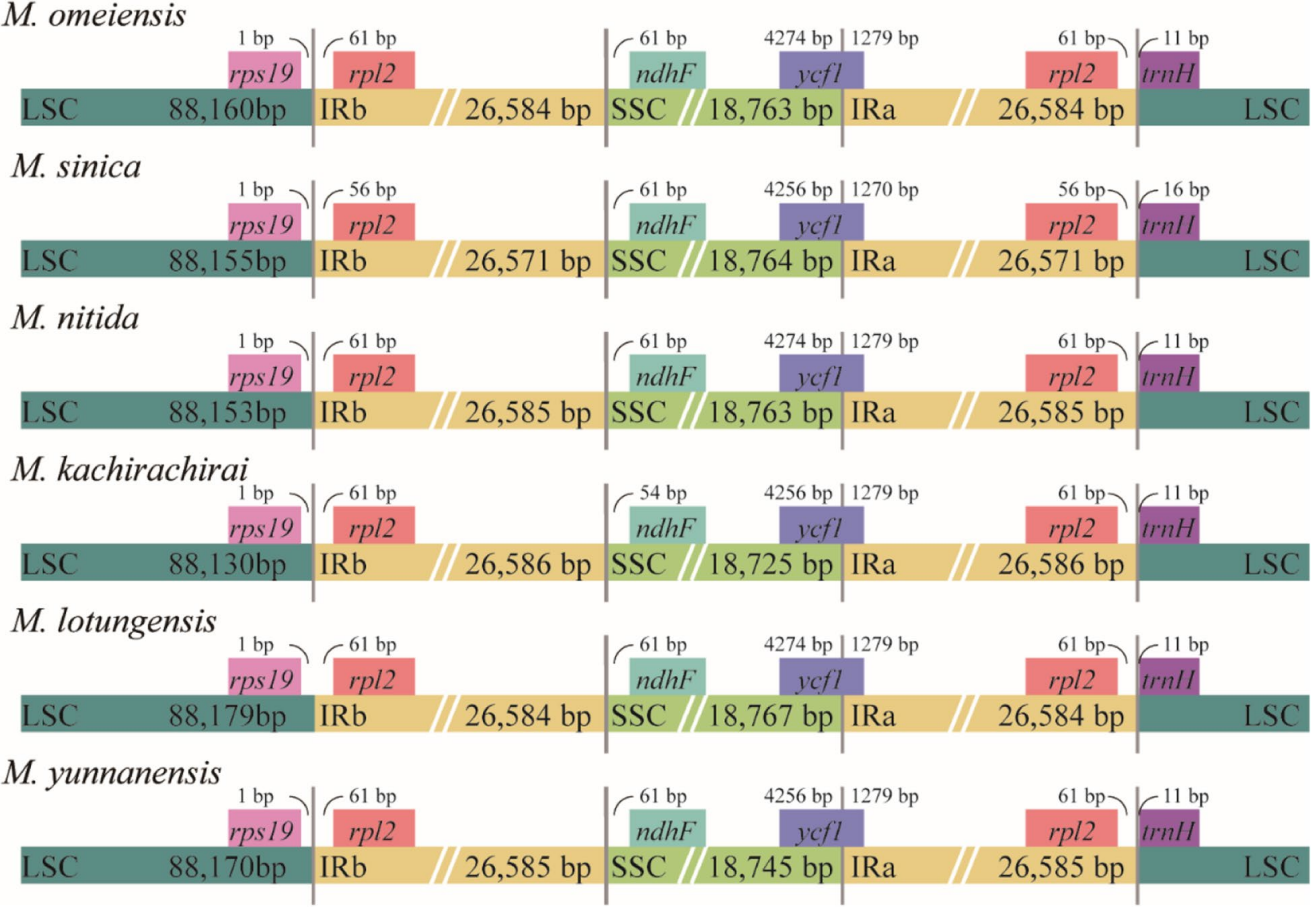
Comparisons of the borders of the large single-copy (LSC), small single-copy (SSC), and inverted repeat (IR) regions among the CPGs of six subgenus *Gynopodium* species. Gaps between the ends of boundaries and adjacent genes were indicated in base pairs (bps) above the main line

Expansions and contractions in the CPGs of six subgenus *Gynopodium* species were visualized using IRscope (Fig. 3). The gene *rps19* and *trnH* were located in the LSC region 1 bp from the LSC/IRb border and 11 bp from the IRa/LSC border. The genes *rpl2* and *ndhF* were located in the IRb and SSC regions, respectively, and differed slightly in their proximity to the border between the IRb and SSC regions. The gene *ycf1* was located between 4,256 and 4,274 bp in the SSC region, and between 1,270 and 1,279 bp in the IRa region. In all CPGs, significant length variations were detected in the LSC and SSC regions; sequences length was more conserved in the IR regions than those in the LSC and SSC regions (Fig. 3 and Table 1).

### Large repeat sequences and Simple sequence repeats (SSRs) analyses

**Fig. 4:**
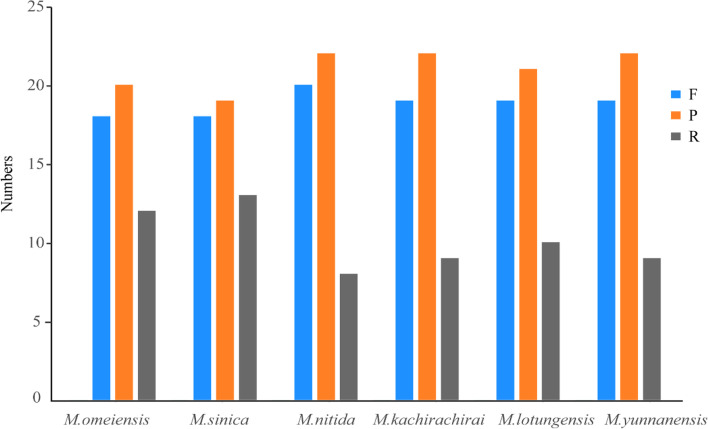
Comparison of the numbers of repeats among the CPGs of six subgenus *Gynopodium* species: *Magnolia omeiensis*, *Magnolia sinica*, *Magnolia nitida*, *Magnolia kachirachirai*, *Magnolia lotungensis*, and *Magnolia yunnanensis*. (F: Forward, P: Palindromic, and R: Reverse repeats)

Large repeat sequences were identified using REPuter software [26]. A total of 300 repeats were identified. Palindromic repeats were the most common repeat sequences, and no complement repeat was found in the CPGs of six subgenus *Gynopodium* species (Fig. 4). Variation was observed in the number of palindromic repeats and reverse repeats among the six CPGs. The lowest number of palindromic repeats (19) was observed in *M. sinica,* followed by *M. omeiensis* (20), *M. lotungensis (21). M. nitida (22), M. kachirachirai* (22), and *M. yunnanensis* (22). The number of reverse repeats was less in *M. nitida, M. kachirachirai*, and *M. yunnanensis* (9) than in *M. lotungensis* (10), *M. omeiensis* (12), and *M. sinica* (13). Among these repeats, nine were over 30 bp and 24 were 20–29 bp; the longest repeat was 39 bp. Over half of the repeats (60%) were located in non-coding regions, and some of the repeats were located in the coding regions of genes, such as *psaA*, *psaB*, *ndhC*, *ycf1*, *ycf2*, *rpoB*, and *rpoC2* (Table S2).

**Fig. 5:**
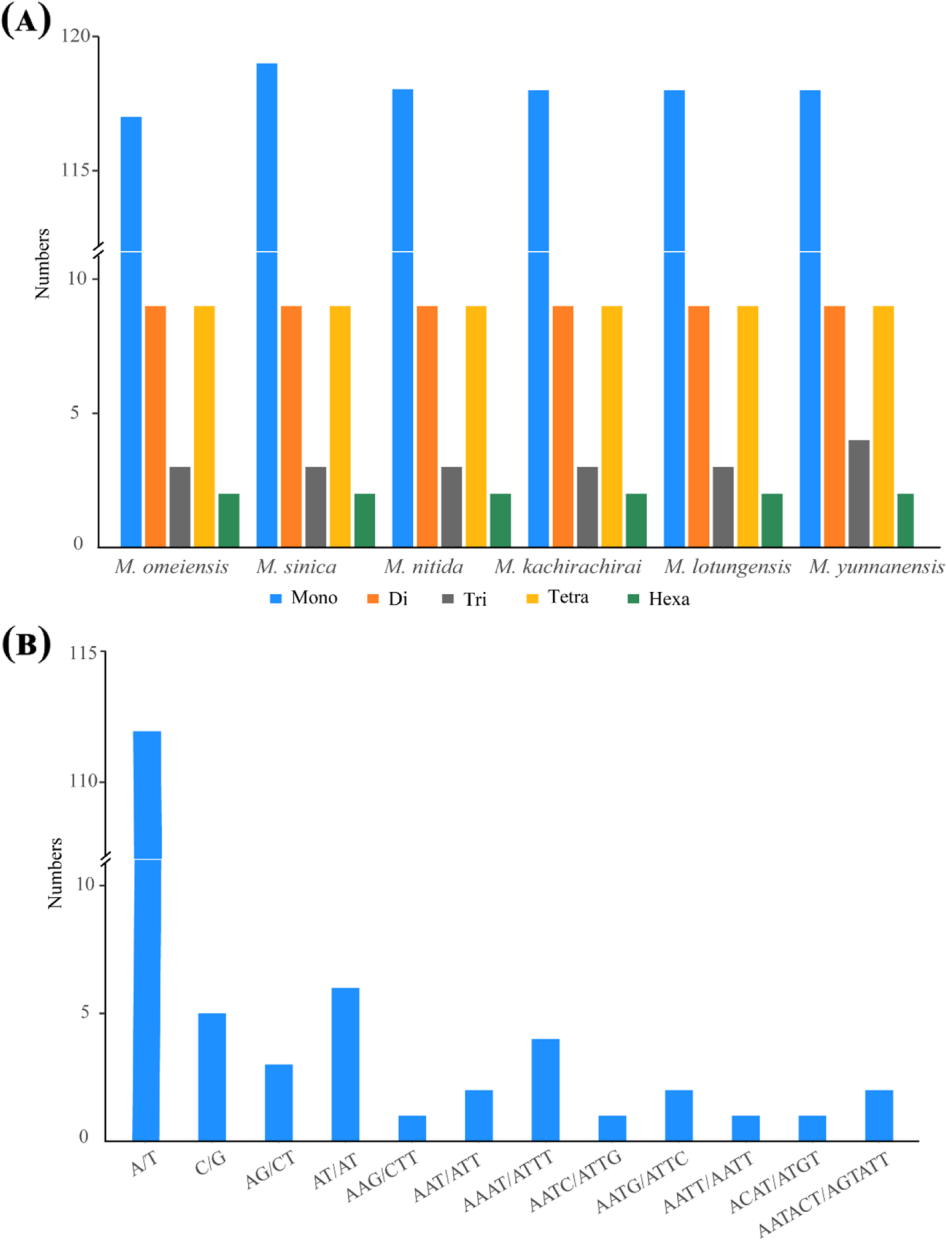
The number of microsatellite loci with different types of repeats (**A**) and repeat units (**B**) detected in the CPGs of six subgenus *Gynopodium* species

A total of 847 SSRs were identified in the CPGs of six subgenus *Gynopodium* species, ranging from 140 to 142 in each species, among which 117–119 were mononucleotides, 9 were dinucleotides, 3–4 were trinucleotides, 9 were tetranucleotides, and 2 were pentanucleotides (Fig. 5a and Table 3). There was no marked variation in the number of SSRs among the six species; however, slight differences were observed in the number of mononucleotides and trinucleotides. Over 80% of SSRs were mononucleotide repeats consisting of 112 A/T repeats and five C/G repeats. All the dinucleotides consisted of multiple copies of AT/TA repeats and AG/CT repeats (Fig. 5b). SSRs were mostly located in intergenic spacer regions (IGS) (69.29%), followed by coding regions (17.86%) and introns (12.86%) (Fig. S2, Table 3). The SSRs in the coding regions were located in 12 protein-coding genes (*rpoC1*, *rpoC2*, *rpoB*, *psbC*, *cemA*, *rps3*, *rps19*, *ndhF*, *ndhD*, *ycf1*, *ycf2*, and *ycf4*) (Table S3). Few SSRs were located in the IR regions (10–12 SSRs); most were located in the LSC region (104–106 SSRs), followed by the SSC region (24–25 SSRs; Table S3).

**Table 3:** The number and location of simple sequence repeats (SSRs) in the subgenus *Gynopodium* species

Species	SSR loci no	mono	di	tri	tetra	penta	Location			Region		
							**IGS**	**Intron**	**CDS**	**LSC**	**IR**	**SSC**
*M. omeiensis*	140	117	9	3	9	2	97	18	25	106	10	24
*M. sinica*	142	119	9	3	9	2	99	18	25	105	12	25
*M. nitida*	141	118	9	3	9	2	98	18	25	105	12	24
*M. kachirachirai*	141	118	9	3	9	2	98	18	25	104	12	25
*M. lotungensis*	141	118	9	3	9	2	98	18	25	105	12	24
*M. yunnanensis*	142	118	9	4	9	2	99	18	25	106	12	24

*mono* mononucleotide, *di* dinucleotides, *tri* trinucleotides, *tetra* tetranucleotide, *penta* pentanucleotide

### Identification of highly variable regions

**Fig. 6:**
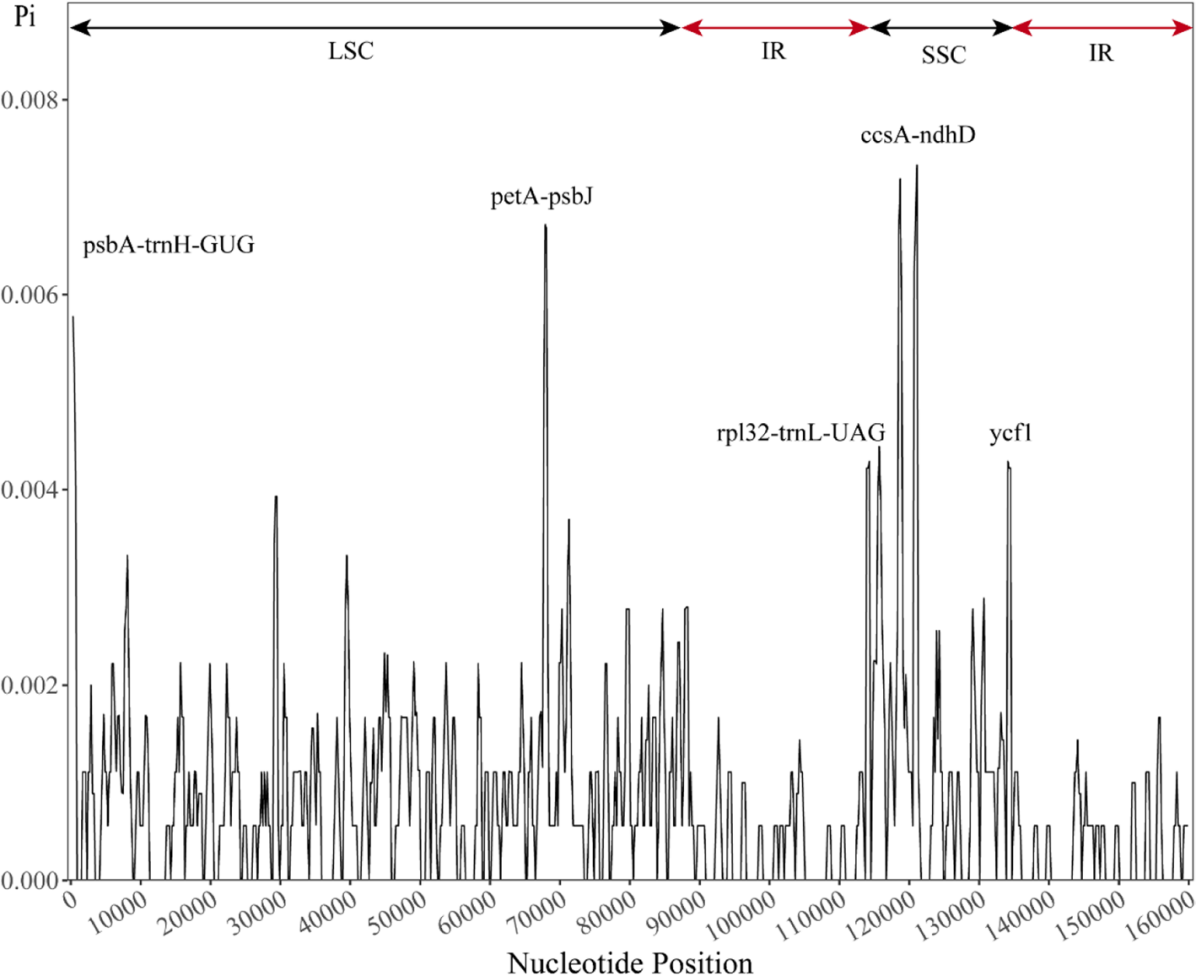
Sliding window test of nucleotide diversity (*Pi*) in the multiple alignments of six subgenus *Gynopodium* species (window length: 600 bp; step size: 200 bp). The X-axis indicates the position of the midpoint of the window; the Y-axis indicates the nucleotide diversity of each window

The nucleotide diversity within a 600-bp window was calculated for all six CPGs, which ranged from 0 to 0.008 (Fig. 6). There were five highly variable regions with *Pi* values greater than 0.004, including the *ycf1* gene and four intergenic regions (*psbA-trnH-GUG*, *petA*-*psbJ*, *rpl32-trnL-UAG* and *ccsA-ndhD*). *Pi* was greatest (0.007) for the intergenic region between *ccsA* and *ndhD*. Highly variable regions were located in the LSC region (2) and SSC region (3); no highly variable region was detected in the IR region (Fig. 6), which reflects similar patterns with structure variability of CPGs. In addition, we evaluated the potential utility of the five highly variable regions. The *rpl32-trnL-UAG* marker (π = 0.007) with the highest discriminatory power can discriminate six haplotypes from the six subgenus *Gynopodium* species (Table 4). The *psbA-trnH-GUG* marker (π = 0.006) with high haplotype diversity can discriminate five haplotypes. Similarly, the marker *petA-psbJ* (π = 0.005), *ccsA-ndhD* (π = 0.007), and *ycf1* (π = 0.004) can discriminate three haplotypes from the six subgenus *Gynopodium* species (Table 4).

**Table 4:** Nucleotide diversity and discriminatory power of the subgenus *Gynopodium* chloroplast markers

Loci	*psbA-trnH-GUG*	*petA-psbJ*	*rpl32-trnL-UAG*	*ccsA-ndhD*	*ycf1*
Number of sequence	6	6	6	6	6
Number of haplotype	5	3	6	3	3
Haplotype diversity	0.93	0.60	1.00	0.73	0.60
*Pi*	0.006	0.005	0.007	0.007	0.004
Polymorphic site	8	9	9	9	7
PI site	4	0	5	7	1
Singleton site	4	9	4	2	6

*Pi* nucleotide diversity, *PI site* Parsimony informative site

### Phylogenetic relationships

**Fig. 7:**
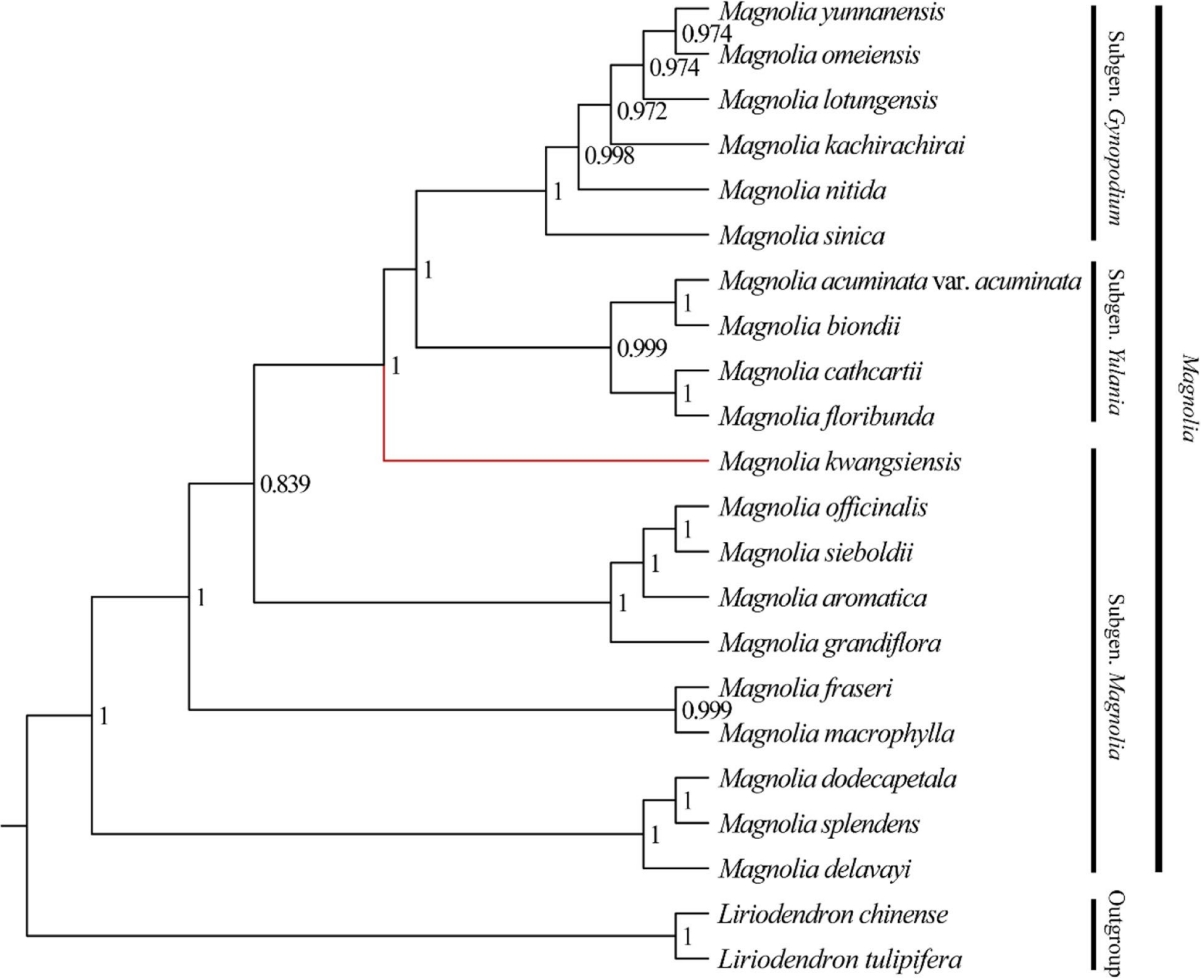
Phylogenetic relationship of Magnoliaceae based on the CPGs of 20 *Magnolia* species and two *Liriondendron* species. The phylogeny was inferred by Bayesian inference. Numbers above the lines indicate the posterior probabilities from the Bayesian inference

Phylogenetic relationships were reconstructed using both ML and BI approaches, based on the whole CPGs of 22 species covering all known sections within Magnoliaceae. Topologies of the ML and BI trees were concordant and confirmed that Magnoliaceae comprised two subfamilies (Liriodendroideae and Magnolioideae), each with one genus (*Liriodendron* and *Magnolia*). Within *Magnolia*, subgenus *Gynopodium* was sister to the subgenus *Yulania* (BS = 100%, PP = 1.00) (Fig. 7). However, due to the non*‐*monophyly of subgenus *Magnolia*, three previously established subgenera in *Magnolia* were not supported (Fig. 7). Subgenus *Gynopodium* should be treated as a section of genus *Magnolia* following Wang et al. (2021) [27]. Within Subgenus *Gynopodium, M. sinica* diverged first (PP = 1, BS = 100), followed by *M. nitida, M. kachirachirai,* and *M. lotungensis* (albeit with relatively low support values), and *M. omeiensis* was sister to *M. yunnanensis* (PP = 0.97, BS = 50) (Fig. 7, Fig. S3).

## Discussion

### Characteristics of the CPGs

The CPGs of most angiosperms varied in size from 120 to 160 kb [16]. Our results indicated that the CPGs of six subgenus *Gynopodium* species are similar in size (ca. 160 kb) and structure (quadripartite circular structure) to other *Magnolia* species [28–30] as well as other higher plants [31]. The total number, order, and composition of genes in the CPGs were highly conserved within subgenus *Gynopodium*, which is also consistent with most *Magnolia* species [32, 33], suggesting a very conserved structure of CPGs of subgenus *Gynopodium*.

The overall GC content has been reported to be associated with the phylogenetic position; specifically, the GC content tends to be higher in early diverged lineages, such as magnoliids [34]. Our results are consistent with these previous findings. Of the six subgenus *Gynopodium* species, the overall GC content of CPGs was approximately 39.3%, which is similar to that of other *Magnolia* species, such as *M. shiluensis* [32]*, M. grandiflora* [35]*,* and *M. zenii* [36] but higher than the average GC content (35%) of most angiosperms [37]. The GC content also varies among different regions of the CPG [34, 38]. IR region (43.2%) contains significantly higher GC content than that of the LSC (38%) and SSC regions (34.3%) (Table S1), which can attribute to the high GC content in the ribosomal RNA (rRNA) genes in IR region (Fig. 1). Identical findings have been reported in other species, such as *Magnolia polytepala* [39], *Magnolia delavayi* [40] and *Datura stramonium* [41].

### Conservatisms of the CPGs

We compared the CPGs of six species within the subgenus *Gynopodium*. The results indicated that the SSC and LSC regions were more divergent than IR regions, and sequences in non-coding regions were more divergent than that in coding regions, which were consistent with previous findings in *Magnolia* species [29] and other flowering plants [42, 43] In this study, we identified six regions presenting significant variations in the CPGs of subgenus *Gynopodium* species, such as five intergenic regions: *trnH*-*psbA*, *rps2*-*rpoC2*, *ycf4*-*cemA*, *petA*-*psbJ*, and *ccsA*-*ndhD*, and one gene *ycf1* (Fig. 2). No major genomic rearrangements or insertions were detected among the six CPGs, which further corroborated the results of recently published studies about Magnoliaceae [27]. Previous studies also found that variation in the size of angiosperms CPGs might be largely driven by length variation in IR regions, intergenic regions, and the number of gene copies [44–46]. The structure of the six CPGs within subgenus *Gynopodium* species was highly conserved; no major expansions or contractions were observed in the IR regions. However, variations in sequence length have been observed in both the LSC and SSC regions, which may drive variations in the size of CPGs within the subgenus *Gynopodium* species, as reported in other species [29, 47, 48].

### Large repeats and simple sequence repeats

Knowledge of genetic diversity within subgenus *Gynopodium* is necessary to develop sustainable conservation management that ensures long-term maintenance of the genetic diversity within these species [3, 49]. Repeat sequences, which are dispersed in CPGs, are an important source of structural variation and play a significant role in genomic evolution [16, 50]. In our study, 300 repeats were identified, of which palindromic repeats were the most common, while complement repeats were missing in CPGs of the subgenus *Gynopodium.* The different number of forward repeats, palindromic repeats and reverse repeats generated the variations of CPGs [41]. Therefore, genetic variation in large repeats can provide useful information for phylogenetic research and population genetics. Previous studies have indicated that repeat sequences are mostly located in the intergenic spacer regions, followed by the coding regions [14, 32]. Our findings are consistent with this general pattern; 61.22-65.31% of the repeats were located in IGS regions, followed by coding regions and introns (34.69-38.38%) (Table S2).

SSRs are useful molecular markers that have been widely used in species discrimination, breeding and conservation, and phylogenetic studies [51–54]. In the CPGs of six subgenus *Gynopodium* species, the number of SSRs located in the LSC and SSC regions accounted for 92.86% of all SSRs, and only ten SSRs were located in the IR region (Table S3). Our findings were consistent with the general pattern of angiosperm that most of the repeats were located in the LSC and SSC regions of CPGs [36, 48]. The SSRs of the CPGs of six subgenus *Gynopodium* species identified in our study provided valuable sources for developing primers of specific SSR loci and a useful tool for species identification.

### Highly variable regions

Highly variable regions provide abundant phylogenetic information and can be used as potential molecular markers to delimit closely related taxa [55]. The *Pi* of highly variable regions within subgenus *Gynopodium* species was lower (< 0.008) compared with previously published values of other species [56, 57] and some of *Magnolia* species [29, 30]. The low genetic diversity of subgenus *Gynopodium* species and other *Magnolia* species, e.g., *Magnolia ashei* may relate to their limited habitat and small populations as threatened species [54, 58, 59].

In the Magnoliaceae, several highly variable regions, such as, *matk*, *ycf1*, *psbA*-*trnH* and *atpB*-*rbcL* have been recognized as potential sites for DNA barcoding [39, 60]. In this study, we recognized five highly variable regions with *Pi* values greater than 0.004, including one gene (*ycf1*) and four intergenic regions (*psbA-trnH-GUG*, *petA*-*psbJ*, *rpl32-trnL-UAG* and *ccsA-ndhD*). The highly variable regions identified here have high discriminatory power to distinguish 6 (*rpl32-trnL-UAG*), 5 (*psbA-trnH-GUG*), 3 (*petA-psbJ*), 3 (*ccsA-ndhD*), and 3 (*ycf1*) plastid haplotypes from six subgenus *Gynopodium* species (Table 4). These regions could be considered as potential barcoding markers for species identification of subgenus *Gynopodium*.

### Phylogenetic relationship

CPGs have shown substantial power in solving phylogenetic relationships among angiosperms [61]. However, it is still controversial regarding the boundaries of the genera of Magnoliaceae [1, 6]. Based on the whole CPGs of 22 species covering all known sections of Magnoliaceae, topologies of the ML and BI trees all supported that Magnoliaceae consisted of two subfamilies Magnolioideae and Liriodendroideae, each with one genus, *Magnolia* and *Liriodendron*, respectively. However, due to the non*‐*monophyly of subgenus *Magnolia*, three previously established subgenera in *Magnolia* were not supported. Our results supported the infrageneric circumscriptions reported by Wang et al. that classified *Magnolia* into 15 clades corresponding to 15 sections and subgenus *Gynopodium* treated as a section of *Magnolia* [27, 62]. And our results also supported merging section *Manglietiastrum* into section *Gynopodium* as reported previously [62, 63].

Although we recovered the phylogenetic relationship within subgenus *Gynopodium*, some of the nodes were poorly supported (Fig. 7). The low nucleotide diversity and nucleotide substitution rate in the CPGs of subgenus *Gynopodium* species and other *Magnolia* species might contribute to the lack of phylogenetic resolution in Magnoliaceae [62, 64, 65]. Consequently, genetic markers from the mitochondrial and nuclear genomes should be developed to reconstruct more robust phylogenies of subgenus *Gynopodium* species.

## Conclusions

We compared the complete CPGs of six subgenus *Gynopodium* species (four newly sequenced and two obtained from previous studies). All CPGs exhibited the typical quadripartite structure of most angiosperms. The number, composition, and order of genes in the CPGs of subgenus *Gynopodium* species were similar to those of other species in the Magnoliaceae. We detected neither major expansions or contractions in the IR region, nor rearrangements or insertions. We identified large repeats, SSRs, and highly variable regions within subgenus *Gynopodium*, getting knowledge of the extremely low genetic diversity in these species. The six highly variable regions identified here will be useful for species delimitation within the subgenus *Gynopodium*. Overall, our findings and genetic resources presented here will facilitate future studies of subgenus *Gynopodium* and aid in species discrimination and conservation strategy development for threatened species in this subgenus.

## Materials and methods

### Plant material, DNA extraction and sequencing

Leaf samples of *M. omeiensis* were collected from mature trees from wild populations on Emei Mountain (Sichuan, China). Leaf samples of *M. nitida* were collected from Nanjing Botanical Garden. Leaf samples of *M. kachirachirai* and *M. sinica* were collected from South China Botanical Garden. The plant materials were identified by Dr. Lei Zhang and the voucher specimens (collection numbers: LiuJQ-2019–123, LiuJQ-2019–168, LiuJQ-2019–050, and ZC-1906–7) were deposited in the herbarium of Sichuan University. The CPGs of 20 species spanning all sections within Magnoliaceae were obtained from the National Center of Bio-technology Information (NCBI, https://www.ncbi.nlm.nih.gov/). *Liriodendron chinense* and *Liriodendron tulipifera* were used as outgroups, and the two CPGs for these species were downloaded from NCBI (Table S4).

Total genomic DNA was extracted from silica gel‐dried leaves using a modified CTAB method [66] and treated with RNase (TransGen, China). The DNA samples were indexed by tags and pooled together in a single lane of a Genome Analyzer (Illumina HiSeq 2000) for sequencing at BGI-Shenzhen. Paired‐end reads (2 × 150 bp) were sequenced, and more than 4.0 Gb of reads were obtained for each sample.

### Assembly and annotation

The raw Illumina reads were first filtered by removing paired-end reads that contained (i) adapter sequences, (ii) more than 10% N bases, and (iii) more than 50% of bases with a Phred quality score less than ten. The filtered reads were then assembled using NOVOPlasty version 4.0 [67] and the complete plastome sequence of *Magnolia biondii* Pamp. (KY085894) as a reference. These assemblies were manually inspected using Geneious Prime version 9.1.8 [68]. The genome was automatically annotated using Plann version 1.1 [69] based on the well-annotated plastome of *M. insignis* Wall. (KY921716). All annotated CPGs were submitted to GenBank (accession numbers: OL631157, OL631158, OL631159, and OL631160). The chloroplast genomes map was generated by OGDRAW version 1.2 [70].

### Comparative analysis of the CPGs of subgenus *Gynopodium* species

The results of the comparative analysis of the CPGs of the six subgenus *Gynopodium* species were visualized using online mVISTA software [71] with the annotated CPG of *M. omeiensis* as the reference in Shuffle-LAGAN mode. Detection of structural variation was conducted using Mauve software [72] with *M. omeiensis* as the reference. The borders of the four different regions among the six CPGs were visualized using IRscope [73].

### Repeat structure and highly variable regions analysis

The online software REPuter [26] was used to identify repeat sequences (forward, reverse, complement, and palindromic) in CPGs with default parameters. Simple sequence repeats were examined using MISA-web [74] with minimal repeat numbers of 8, 5, 4, 3, 3, and 3 for mono-, di-, tri-, tetra-, penta-, and hexa-nucleotide repeats, respectively. To identify highly variable regions, polymorphic sites and nucleotide diversity (*Pi*) in the six MAFFT-aligned CPGs were assessed using a sliding window analysis in DNAsp v6.12.03, with a 200-bp step size and a 600-bp window length [75]. Regions in the CPGs with numbers of polymorphic sites greater than the sum of the average and double the standard deviation were considered highly variable regions [76]. Then we estimated the number of haplotypes, haplotype diversity, parsimony informative sites, and singleton sites to detect the discriminatory power of highly variable regions using DnaSP v6.12.03 [75].

### Phylogenetic analysis

Phylogenies were reconstructed using maximum likelihood (ML) and Bayesian inference (BI) analyses with the complete CPGs of 20 *Magnolia* species and two *Liriodendron* species (Table S4). ML analysis was conducted in RAxML [77] using the GTRGAMMA model and 1000 bootstrap (BS) replicates. BI analysis was conducted in Mrbayes v 3.2.6 [78], with four independent Markov chain Monte Carlo analysis runs for 1,000,000 generations each. PartitionFinder was used to determine the optimal partitioning scheme [79]. Priors were set to default values, and trees were sampled every 1,000 generations, with the first 25% discarded as burn-in. The consensus tree was calculated from trees sampled after reaching likelihood convergence, and the posterior probabilities (PPs) of the tree nodes were calculated.

## Supplementary Information

**Additional file 1: Figure S1.** Alignment of the CPGs of sixsubgenus *Gynopodium* species by Mauve. The *Magnolia omeiensis* genome (the reference genome) is shown at the top. Color bars indicate locally collinear blocks, and connecting lines indicate correspondingblocks across genomes.

**Additional file 2: Figure S2.** Proportion of simplesequence repeats in the inverted repeat (IR), large single-copy (LSC), and small single-copy (SSC) regions (A) and in the intergenic spacer (IGS),coding (CDS), and intron regions(B).

**Additional file 3: Figure S3.** Phylogenetic relationship of the family Magnoliaceae (20 *Magnolia* species and two *Liriondendron* species) based on the CPGs.Phylogenies were inferred by maximum likelihood analysis. Numbers above thelines indicate the bootstrap values from the maximum likelihood analysis.

**Additional file 4: Table S1.** GC content of the CPGs of the six subgenus *Gynopodium* species.

**Additional file 5: Table S2.** List of large repeat sequences in the CPGs of six subgenus *Gynopodium* species.

**Additional file 6: Table S3.** List of SSRs in the CPGs of six subgenus *Gynopodium* species.

**Additional file 7: Table S4.** Accession numbers of the CPGs used in the phylogenetic analysis.

## Data Availability

All annotated chloroplast genomes have been deposited in GenBank (https://www.ncbi.nlm.nih.gov/genbank/), and accession numbers are provided in Additional file 7. Other data generated or analyzed in our study are included in the additional files.

## Abbreviations

CPG: Chloroplast genome
IGS: Intergenic spacer regions
IR: Inverted repeat
LSC: Large single-copy
SSC: Small single-copy
CDS: Coding sequence
SSRs: Simple sequence repeats
GC: Guanine-cytosine
PCG: Protein-coding gene
BI: Bayesian inference
ML: Maximum Likelihood
IUCN: International Union for the Conservation of Nature
