# Supplementary material for: Comparative analysis of the complete chloroplast genomes of six threatened subgenus Gynopodium (Magnolia) species
Source: BMC Genomics. 2022 Oct 20;23:716. doi: 10.1186/s12864-022-08934-6 (PMC9583488; doi:10.1186/s12864-022-08934-6)
Supplement: Supplementary file 1 — Additional file 1: Figure S1. Alignment of the CPGs of sixsubgenus Gynopodium species by Mauve. The Magnolia omeiensis genome (the reference genome) is shown at the top. Color bars indicate locally collinear blocks, and connecting lines indicate correspondingblocks across genomes. [file 12864_2022_8934_MOESM1_ESM.docx]

**Additional file 1: Figure S.** Alignment of the CPGs of six subgenus *Gynopodium* species by Mauve. The *Magnolia omeiensis* genome (the reference genome) is shown at the top. Color bars indicate locally collinear blocks, and connecting lines indicate corresponding blocks across genomes.

**Figure S1**

**
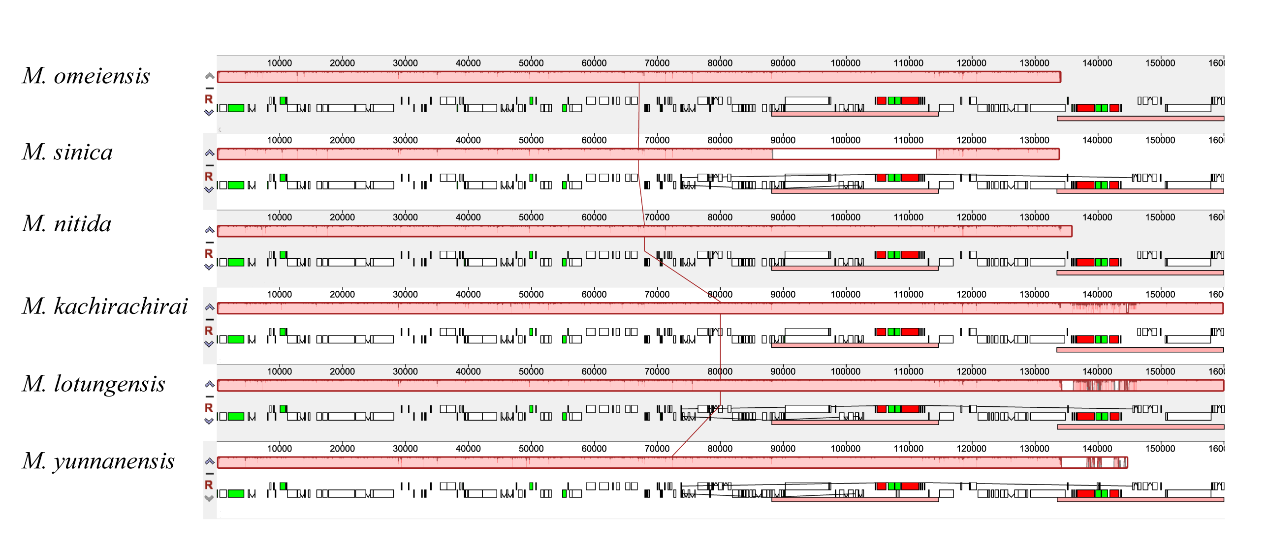
**
