# Supplementary material for: Comparative analysis of the complete chloroplast genomes of six threatened subgenus Gynopodium (Magnolia) species
Source: BMC Genomics. 2022 Oct 20;23:716. doi: 10.1186/s12864-022-08934-6 (PMC9583488; doi:10.1186/s12864-022-08934-6)

**Additional file 2: Figure S2.** Proportion of simple sequence repeats in the inverted repeat (IR), large single-copy (LSC), and small single-copy (SSC) regions **(A)** and in the intergenic spacer (IGS), coding (CDS), and intron regions**(B)**.

**Figure S2**


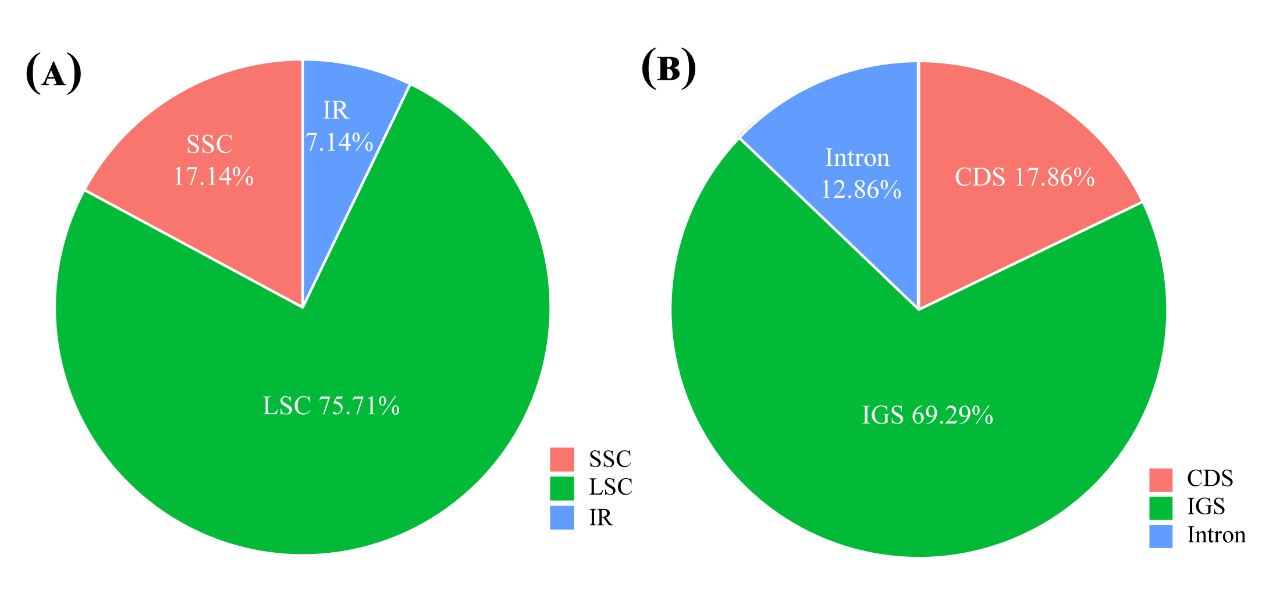

Supplement: Supplementary file 2 — Additional file 2: Figure S2. Proportion of simplesequence repeats in the inverted repeat (IR), large single-copy (LSC), and small single-copy (SSC) regions (A) and in the intergenic spacer (IGS),coding (CDS), and intron regions(B). [file 12864_2022_8934_MOESM2_ESM.docx]
