# Supplementary material for: Comparative analysis of the complete chloroplast genomes of six threatened subgenus Gynopodium (Magnolia) species
Source: BMC Genomics. 2022 Oct 20;23:716. doi: 10.1186/s12864-022-08934-6 (PMC9583488; doi:10.1186/s12864-022-08934-6)
Supplement: Supplementary file 3 — Additional file 3: Figure S3. Phylogenetic relationship of the family Magnoliaceae (20 Magnolia species and two Liriondendron species) based on the CPGs.Phylogenies were inferred by maximum likelihood analysis. Numbers above thelines indicate the bootstrap values from the maximum likelihood analysis. [file 12864_2022_8934_MOESM3_ESM.docx]

**Additional file 3: Figure S3.** Phylogenetic relationship of the family Magnoliaceae (20 *Magnolia* species and two *Liriondendron* species) based on the CPGs. Phylogenies were inferred by maximum likelihood analysis. Numbers above the lines indicate the bootstrap values from the maximum likelihood analysis.

**Figure S3**

**
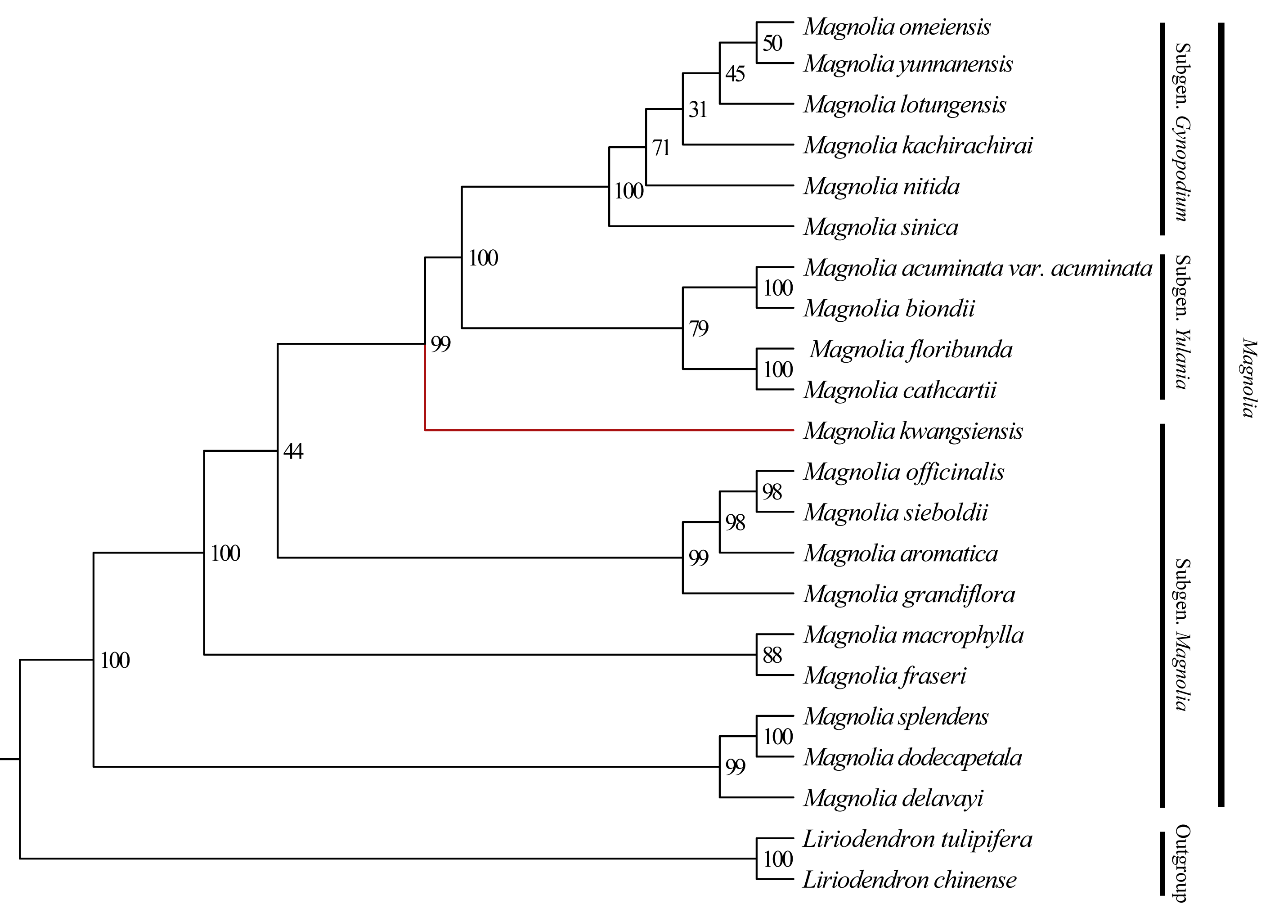
**
